# Optic Nerve Sheath Diameter Measurement During Diabetic Ketoacidosis: A Pilot Study

**DOI:** 10.5811/westjem.2016.6.29939

**Published:** 2016-07-25

**Authors:** Kelly R. Bergmann, Donna M. Milner, Constantinos Voulgaropoulos, Gretchen J. Cutler, Anupam B. Kharbanda

**Affiliations:** * Children’s Hospitals and Clinics of Minnesota, Department of Pediatric Emergency Medicine, Minneapolis, Minnesota; † Children’s Hospitals and Clinics of Minnesota, McNeely Pediatric Diabetes Center and Endocrinology Clinic, Minneapolis, Minnesota

## INTRODUCTION

Diabetic ketoacidosis-related cerebral edema (DKA-CE) occurs in up to 1% of children with type 1 diabetes (T1D),^1^ with approximately 20% displaying neurologic symptoms at presentation.^2^,^3^ Similarly, up to 54% have a mild form of subclinical DKA-CE identified by extracellular fluid measurements on magnetic resonance imaging (MRI), which is associated with alterations in neuronal function and cerebral injury on MR spectroscopy.^2^–^6^ This suggests that DKA-CE occurs along a continuum, ranging from asymptomatic imaging changes, mild neurologic injury, to cerebral herniation and death.

DKA-CE is currently assessed by clinical symptoms and advanced imaging techniques, which have significant cost and limitation. Computed tomography (CT) uses ionizing radiation and can miss nearly 40% of children who ultimately develop DKA-CE.^7^ MRI is time-consuming, often requires sedation, and may not be available at some centers. Moreover, both CT and MRI require moving critically ill patients out of the intensive care setting. In contrast, point-of-care ocular ultrasound is a relatively low cost, accessible imaging technique that may have the potential to promptly identify DKA-CE through measurement of the optic nerve sheath diameter (ONSD).

The sonographic appearance of the ONSD correlates with elevations in intracranial pressure (ICP), which is thought to be due to cerebrospinal fluid accumulation between the dura and optic nerve with the subsequent appearance of swelling.^8^ ONSD measurement has been used to accurately detect increased ICP in a number of clinical situations,^8^–^10^ including pediatric head trauma^11^ and hydrocephalus.^12^,^13^ This method has also been validated in adults using direct measurements of ICP via intrathecal infusion tests,^14^ lumbar puncture,^15^ and intracranial monitoring.^16^

The point at which patients actually progress to subclinical DKA-CE and the minimum duration of preceding symptoms has not been delineated.^7^ Furthermore, it is currently unclear how the ONSD changes during T1D-related illness or whether ONSD measurement can discriminate stages along the continuum of DKA-CE development, particularly among those with subclinical DKA-CE or those without obvious neurologic symptoms. Therefore, we sought to perform a pilot study among patients with T1D and DKA to determine how ONSD measurements vary during T1D-related illness, and the potential of this tool for discrimination of subclinical DKA-CE. We hypothesized that mean binocular ONSD would increase with illness severity, and would differ among children with DKA, T1D with hyperglycemia, and well-controlled T1D. The association of mean binocular ONSD with DKA-CE risk factors was also examined.

## METHODS

### Study Design

This was a cross-sectional study of pediatric patients aged 7–18 years with T1D conducted from March 2014 to May 2015. This study was registered with clinicaltrials.gov (NCT02130180) and approved by the hospital’s institutional review board. Informed consent and assent were obtained from all parents and patients, respectively.

A convenience sample of patients was enrolled at a tertiary children’s hospital with an annual emergency department (ED) census of 90,000. The hospital’s endocrinology clinic is a regionally recognized center for pediatric diabetes education and management, with a T1D census of approximately 1,860 patients. Patients were enrolled in each of three study groups: 1) well-controlled T1D, 2) T1D with hyperglycemia, and 3) DKA. We defined well-controlled T1D as having a no documented hemoglobin A1C >8% and no previous episodes of DKA other than at the time of T1D diagnosis. DKA was defined as hyperglycemia ≥200 mg/dL, venous pH <7.30 and/or bicarbonate level <15 mmol/L, and either positive urine or serum ketones.^17^,^18^ Patients with hyperglycemia ≥200 mg/dL but not meeting other criteria for DKA were included in the T1D with hyperglycemia group. We excluded patients if they had underlying neurologic conditions predisposing to changes in ICP (hydrocephalus, ventriculoperitoneal shunt, Chiari malformation, pseudotumor cerebri, brain tumor), previously documented increases in intraocular pressure, history of type 2 diabetes mellitus, hyperosmolar hyperglycemic nonketotic state or insulin prior to transfer. Given concerns about the potential confounding relationship between larger intravenous (IV) fluid amounts and cerebral edema development, we excluded patients who received >10 mL/kg of IV fluid prior to transfer.

Well-controlled T1D patients were screened for inclusion criteria using the electronic medical record and enrolled during routine follow up in the endocrinology clinic. Patients with T1D with hyperglycemia or DKA were screened at ED triage, and enrolled after eligibility criteria were confirmed and within four hours of arrival to the ED. All children with T1D with hyperglycemia and DKA were treated according to our institution’s hyperglycemia and DKA guideline. This included routine laboratory testing, IV fluid administration, and insulin as determined by the treating physician. Patients were not included if previously enrolled.

The enrolling sonographer collected demographic and medical history via a structured case report form that included information on presenting signs and symptoms, length of symptoms (polyuria, polydipsia, weight loss, vomiting), history of T1D and number of previous DKA episodes, IV fluid and insulin received, treatments administered for DKA-CE, and time from ED registration to ultrasound measurement. The electronic medical record was abstracted to obtain laboratory values (pH, pCO_2_, bicarbonate level, blood glucose, sodium, potassium, blood urea nitrogen, creatinine, serum and urine ketones, hemoglobin A1C), confirm historical information, and review hospital admission and follow-up clinic data (hospital course, results of imaging studies, follow up clinic weights). Percent dehydration was calculated from the weight change from the ED to clinic follow up within one month of hospital discharge. We then entered data into Excel (Microsoft, Redmond, WA).

Missed eligible patients were identified and the electronic medical record was abstracted to obtain available demographic and clinical parameters in order to ascertain selection bias.

### Ultrasound Technique

Sonographers performing ocular ultrasound included a pediatric emergency medicine fellow and pediatric endocrinologist. Ocular ultrasound was performed using a SonoSite M-Turbo ultrasound machine (SonoSite Inc., Bothell, WA) with a 13–6 MHz linear array transducer. Sonographers attended an intensive one-day ultrasound course that included specific training on ONSD measurement. The ultrasound technique was further refined in two separate training sessions using healthy volunteers. Study sonographers were not responsible for the ED care of enrolled patients but were aware of laboratory inclusion criteria in order to assign study group status, and were therefore un-blinded to ultrasound results.

**Figure 1:**
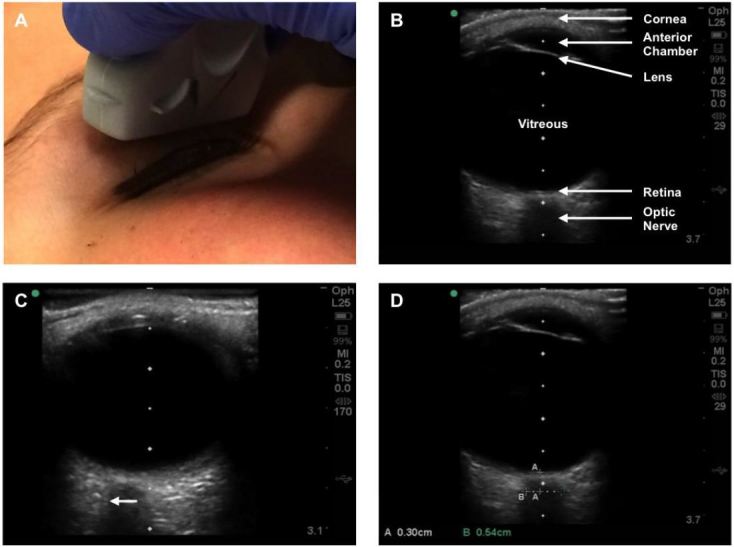
Ocular ultrasound was performed in the transverse plane using a linear transducer to identify the optic nerve (A and B). The nerve sheath was included in optic nerve sheath diameter (ONSD) measurements when visualized (C, arrow) and measurements were taken at a depth of 3 mm posterior to the globe (D).

Images were obtained according to standard technique using the anterior transbulbar approach to image the ONSD in an axial plane along the visual axis (Figure 1).^19^,^20^ Ultrasound gel was placed over the transducer, which was then positioned over a closed eyelid with the patient in a supine position. Patients were instructed to direct their gaze to the midline, and the probe was manipulated as needed to best visualize the optic nerve. Images were then captured and ONSD measurements (mm) were taken directly on the ultrasound machine at a depth of 3 mm posterior to the globe. Previous studies and postmortem investigation have shown that this is the optimal position to detect ONSD swelling,^9^,^21^,^22^ and subsequent studies have established abnormal values associated with elevated ICP as >4.5 mm in children >1 year and >5 mm in adults >18 years.^9^,^13^,^23^ In keeping with standard technique, the nerve sheath was included in measurements when present.^19^,^20^ Images were saved and exported to a secure USB, and the process repeated for the patient’s other eye.

### Statistical Analysis

We analyzed data using SPSS Statistics (version 22; IBM, Armonk, NY). Mean binocular ONSD was the primary outcome measure. Numeric ONSD measurements were obtained and classified as normal or increased in size according to established reference values, where >4.5 mm was considered the upper limit of normal in patients aged 1–18 years.^8^,^13^,^23^ Our secondary outcome was interrater reliability. An additional ocular ultrasound was performed on a subset of patients by the second study sonographer who was blinded to the clinical status and initial ultrasound results. We also examined the association of mean binocular ONSD with DKA-CE risk factors.

We used descriptive statistics to compare patient characteristics with numerical data presented as medians with interquartile range (IQR). We assessed data for normality. The Mann-Whitney U test was used to compare median values between two groups, and one-way ANOVA or the Kruskal-Wallis test was used to compare values between three groups for parametric or non-parametric variables, respectively. We compared categorical variables using χ^2^ and Fisher’s exact test. Missed eligible patients were analyzed for selection bias.

We used multiple linear regression to determine if risk factors were independently associated with mean binocular ONSD. Explanatory variables in the model included factors that may influence the ONSD or development of DKA-CE, including estimated percent dehydration, length of symptoms (days), total amount of IV fluid received, time to ultrasound, time from IV fluid administration to ultrasound, and laboratory values at presentation (pH, pCO_2_, bicarbonate, blood urea nitrogen). Total IV fluid received was separated into three categorizes (≤10 mL/kg, 10–20 mL/kg, and ≥20 mL/kg) and included prehospital and ED fluids received. Missing values were imputed in the regression model using the minimum and maximum for each variable. We excluded well-controlled T1D patients from regression analysis, as this group did not have laboratory investigations aside from hemoglobin A1C. Sensitivity analysis was performed using only images acquired by the pediatric endocrinologist, patients with symptoms ≤24 hours, and those with severe DKA (pH <7.1).^18^ We assessed interrater reliability testing of ONSD measurements between sonographers using the raw percent agreement and the intraclass correlation coefficient.

Previous literature,^12^,^13^,^23^ has shown a mean difference in ONSD ranging from 1–2.8 mm when comparing asymptomatic to symptomatic children, and a standard deviation of approximately 0.36 mm. Given that we were interested in children without obvious neurologic symptoms, we designed our study to detect a lower between group difference in mean ONSD and set this at >0.1 mm, yielding an effect size of approximately 0.3. We therefore aimed to enroll 36 patients in each of three study groups in order to maintain an α=0.05 and power of 0.80.

## RESULTS

**Figure 2:**
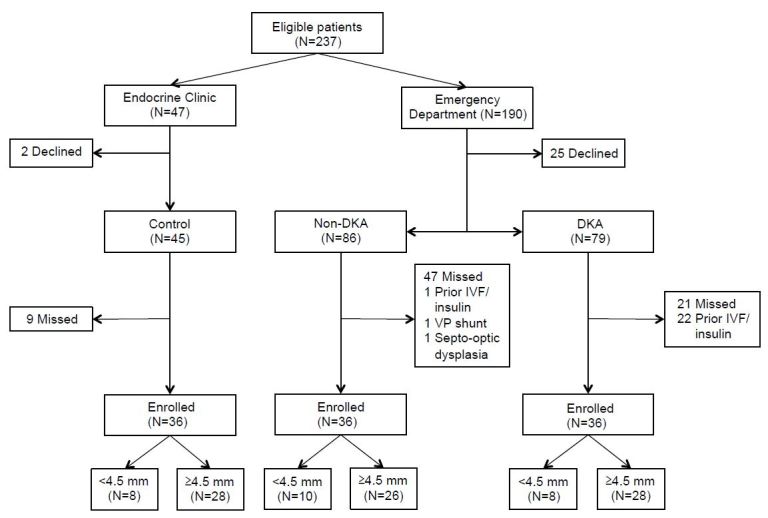
Study flow chart. ONSD measurements were obtained in a total of 108 patients, enrolled in the Endocrine Clinic (controls) or Emergency Department (DKA and non-DKA). Missed eligible patients and those meeting exclusion criteria were tracked throughout the study. A sonographic ONSD measurement ≥4.5 mm was considered enlarged. *DKA*, diabetic ketoacidosis; *IVF*, intravenous fluid; *VP*, ventriculoperitoneal

Study enrollment flow is shown in Figure 2. Demographics were similar among groups (Table 1). African-American children tended to present with DKA more often than white patients, although this difference was not statistically significant. No patients had clinically overt DKA-CE. All but one patient had a Glasgow Coma Scale of 15 at presentation (1 patient with 14).

ED characteristics are shown in Table 2. No patients with T1D with hyperglycemia received IV fluid prior to transfer compared to five patients with DKA (<10 mL/kg). There was no statistical difference in length of symptoms, amount of IV fluid received, or number of patients who received insulin. Among those that received insulin in the ED, all were treated with IV fluid prior to insulin administration according to institutional protocol. Patients with T1D with hyperglycemia had significantly lower median percent dehydration compared to those with DKA (p=0.01). Median sodium, potassium, glucose, blood urea nitrogen, and hemoglobin A1C were similar across groups. Patients with DKA had significantly lower pH, bicarbonate, and pCO_2_. No patients were treated with sodium bicarbonate, mannitol, 3% saline, or steroids, and no patients had neuroimaging.

Available demographics and ED characteristics were largely similar among missed eligible compared to enrolled patients (Table 4). A higher proportion of missed eligible patients with DKA received insulin in the ED compared to enrolled DKA patients (p=0.03). The majority of eligible patients with well-controlled T1D were enrolled and therefore not analyzed.

Seventy-nine (73%) ultrasound examinations were performed by the pediatric emergency medicine fellow and 29 (27%) by the pediatric endocrinologist. Time to ultrasound measurement was similar between groups, although time from IV fluid administration to ultrasound was significantly different between groups (p=0.02; Table 3). The between group difference in mean ONSD (mm) was not significant (p=0.79). Sensitivity analysis using only images acquired by the pediatric endocrinologist showed similar ONSD results among well-controlled T1D (median 5.5, IQR [5.0–6.0]), T1D with hyperglycemia (median 5.4, IQR [4.8–6.2]), and DKA (median 4.8, IQR [4.5–5.9]). There was no difference in the between group mean ONSD when including only patients with severe DKA (n=10, median 6.1, IQR [4.6–6.4]) (p=0.39). There was no difference in mean ONSD when including only T1D with hyperglycemia (n=9, median 4.95, IQR [4.3–5.4]) and DKA (n=9, median 5.20, IQR [4.75–5.83]) patients with symptoms ≤24 hours (p=0.45).

No variables in our regression model were significantly associated with mean ONSD. Four patients (5.5%) did not have a blood urea nitrogen level drawn, and four (5.5%) patients did not have follow-up clinic weights within one month of discharge to calculate estimated percent dehydration. No variables were significantly associated with mean ONSD after imputation using the minimum and maximum values in place of missing variables.

Nine (8.3%) patients had two independent ultrasound examinations performed by the separate study sonographer for internal reliability. The raw agreement between sonographers was 88.9% and the intraclass correlation coefficient was 0.71.

## DISCUSSION

In our study population, ONSD measurements did not vary significantly based on T1D-related illness severity and were not independently associated with presenting laboratory parameters, known DKA-CE risk factors, or time to ultrasound.

Early identification of those at risk for DKA-CE continues to be a challenge despite multiple known risk factors^1^,^24^,^25^ Recent investigation suggests that impaired cerebral autoregulation may lead to vasogenic edema formation.^4^,^5^,^26^–^28^ This has also been supported by measurement of middle cerebral artery flow velocity via transcranial doppler ultrasonography.^29^,^30^ However, ultrasound measurement of middle cerebral artery flow is difficult to reliably reproduce.^31^ Hansen et al. recently investigated ONSD measurements among children with DKA, but their evaluation was limited to only seven patients.^32^

ONSD measurement is a rapid and non-invasive imaging technique that has been used to detect increased ICP in a number of clinical conditions.^8^,^10^–^16^,^23^,^33^–^39^ Sensitivity and specificity have ranged from 83–100% and 84–100% using direct measurement of cerebrospinal fluid pressure or CT as a gold standard reference,^15^,^16^,^33^,^34^,^36^,^37^,^39^ but have been reported to be as low as 75% and 44%, respectively.^35^ In a recent meta-analysis of 12 studies including 478 adults and children, sensitivity and specificity for detecting CT findings associated with increased ICP were 95.6% and 92.3%, respectively.^40^ The pooled OR was 319.3 (95% CI [79–1290]). These findings suggest that measurement of the ONSD accurately identifies elevated ICP.

Investigation using pediatric ophthalmologist or radiologist-performed sonography has shown a significant difference in mean ONSD among children with elevated ICP compared to controls or an asymptomatic baseline measurement.^9^,^10^,^12^,^13^ However, pediatric emergency physician-performed ultrasound studies have shown sensitivities and specificities ranging from 61–83% and 22–38%, respectively, when compared to CT or direct cerebrospinal fluid pressure measurement.^8^,^38^ These studies included children with various etiologies of symptoms, many of whom had ventriculoperitoneal shunt malfunction and obstructive hydrocephalus, which may lead to impaired cerebrospinal fluid circulation and therefore an underestimate of ICP by ONSD measurement and decreased test sensitivity.^41^ Moreover, previous research in children with shunt malfunction has indicated that increased ICP may be present without papilledema,^42^,^43^ which appears sonographically as an increased ONSD with optic disc cupping.

We were able to demonstrate good interrater reliability between sonographers after completion of a structured emergency ultrasound course with further refinement of skills in two separate scanning sessions. This is similar to previous data reported by Le et al, who demonstrated good interrater agreement of ONSD measurements obtained by pediatric emergency medicine fellow/attending compared to a pediatric ophthalmologist (κ=0.64) and ophthalmic sonographer (κ=0.52).^8^ Good to excellent agreement has also been demonstrated among adult emergency medicine resident/attending compared to CT (intraclass correlation coefficient 0.9; 95% CI [0.88–0.93]),^44^ and in studies using MRI^45^ and postmortem specimens.^21^

In our study, the ONSD was measured in an axial plane along the visual axis, which is considered standard technique.^19^,^20^ However, alternative imaging techniques exist. The most common is the coronal approach, which can be performed over the superior lid or infraorbitally, and involves placing the probe along the lateral orbit directed medially.^46^,^47^ The visual axis technique has shown similar ONSD measurements when compared to the infraorbital coronal approach.^46^ Conversely, Blehar et al found that ONSD measurements were significantly larger when measured by the visual axis compared to coronal technique, although these findings were limited to a sample of only 27 patients.^47^ It has been speculated that the visual axis technique may lead to larger ONSD measurements due to potential shadowing along the optic nerve sheath and therefore producing a larger diameter,^47^,^48^ although this has not been studied in children. Additionally, some suggest performing measurements in at least two planes, such as axial and sagittal, to fully characterize pathologic findings.^19^,^20^ The use of single vs. bi-plane imaging for ONSD measurement has not been studied and was therefore not employed in our investigation.

While we found no difference in ONSD measurements between groups, all groups displayed mean measurements above the upper limit of normal for age. It may be that children with T1D have a fundamentally different baseline ONSD, although this has not been systematically evaluated. Our findings may also be explained by our measurement of the ONSD via the axial plane along the visual axis, or by assessment at one point in time rather than obtaining serial measurements during the course of treatment. Previous investigation among adults undergoing intrathecal infusion testing showed that the average cerebrospinal fluid pressure threshold to produce ONSD dilation was 22 mmHg (range 15–30 mmHg), and that ONSD measurements were directly correlated with cerebrospinal fluid pressure above an individual patient’s threshold until a “saturation” point was reached when no further dilation occurred.^14^ These findings suggest that changes in ICP may go undetected by ONSD measurement when ICP is low or normal, and that serial ultrasound measurements may be more useful than single scans once ICP has become elevated above an individual patient’s threshold. This is also supported by previous investigation showing that 20–40% of patients with clinically overt DKA-CE have no signs of edema on initial cranial CT, with subsequent CTs often showing edema and/or hemorrhage.^7^,^49^ Hansen et al recently studied seven children with DKA at various time points and noted that, although no significant difference in effect size, three out of seven patients developed ONSD measurements changes ≥0.3 mm during treatment.^32^ Although we did not compare ONSD values to direct measures of ICP in our study, cerebrospinal fluid pressure may not have been high enough at our data collection point to produce significant ONSD changes reflective of subclinical DKA-CE.

Several aspects of our study are worth further mention. While previous research suggested that IV fluid replacement may precipitate DKA-CE,^50^,^51^ recent investigation has shown that the rate and volume of IV fluid may not substantially contribute.^24^,^26^,^52^ In our study, both treatment groups received ≤10 mL/kg of IV fluid prior to transfer, with no difference in total IV fluid amount received. Although time from IV fluid administration to ultrasound was significantly different between groups, we believe this likely reflects the difficulties in obtaining IV access in severely ill children, which is supported by our finding that percent dehydration was higher among those with DKA. Moreover, ONSD values were not affected by IV fluid amount, time from IV fluid administration to ultrasound, length of symptoms, or other known DKA-CE risk factors in our regression model. It should be noted, however, that our study was powered for our primary outcome to detect a difference in mean ONSD rather than multiple regression. A post-hoc power analysis of our regression model, with nine predictor variables and an observed R^2^ of 0.39, demonstrated an observed power of 0.63.

There was a higher proportion of African-American patients with DKA in our sample compared to White or Hispanic patients. Although this difference was not statistically significant, this may represent a clinically significant difference and is in keeping with recent literature showing that African-American children with T1D experience poorer glycemic control and more episodes of DKA compared to White or Hispanic children.^53^ Finally, although DKA-CE is more common in younger children,^1^,^54^ we excluded patients <7 years for assent purposes.^55^

## LIMITATIONS

Point-of-care ultrasound requires training and is operator dependent, which may limit generalizability. We did not assess for the presence of secondary signs that may indicate elevated ICP, such as optic disc elevation, or the “crescent” or “doughnut” sign,^19^,^56^ and we did not perform analysis of accumulated ultrasonography experience given the number of patients. However, measurement of the ONSD, as was performed in this study, is considered standard for evaluation of elevated ICP. We enrolled a convenience sample of patients due to the limitations of sonographer availability, which resulted in a number of missed eligible subjects. Nonetheless, missed eligible patients were largely similar to those enrolled, making this unlikely to have affected our results (Table 4). Study sonographers enrolled patients prior to examinations and were therefore not blinded to clinical status. Our study would have benefited from comparison of ONSD measurements to measures of ICP, such as opening pressure or the apparent diffusion coefficient via MRI. However, such evaluation was beyond the scope of our pilot investigation.

We were unable to enroll any patients with clinically overt DKA in our convenience sample, although one patient had a GCS of 14. In addition, excluding patients who received >10 mL/kg of IV fluid or insulin prior to transfer may have resulted in exclusion of patients likely to develop DKA-CE. However, the point at which progression to subclinical or clinically overt disease occurs, and the minimum duration of preceding symptoms has not been delineated.^7^ Therefore, the aim of this pilot investigation was to assess how the ONSD changed during episodes of DKA, particularly among patients without obvious neurologic symptoms, and therefore inform larger studies that may evaluate patients with known subclinical or clinically overt disease. For these reasons, we designed our study with more rigorous inclusion/exclusion criteria. Although accounting for more acute symptoms prior to presentation may have allowed us to distinguish patients at higher risk of DKA-CE, as neurologic symptoms often develop precipitously,^2^,^24^ sensitivity analysis among patients with symptoms ≤24 hours showed no difference in ONSD.

Although no previous studies have evaluated the influence of insulin or IV fluid on ONSD measurement, it is unclear if these therapies significantly alter cerebrospinal fluid volume or pressure, and therefore the ONSD, during treatment of DKA. This is unlikely to have substantially affected our results given that the proportion of patients receiving insulin, total amount of IV fluid received, and time to ultrasound were similar between groups. A significantly higher proportion of missed eligible DKA patients received insulin in the ED, which suggests that we may have enrolled less ill patients in our study. Yet, this is unlikely given that missed eligible DKA patients had similar pH, bicarbonate, glucose, blood urea nitrogen, and percent dehydration compared to enrolled patients.

Our study highlights several key issues for future research efforts. First, multi-center investigation will be required to capture patients with DKA-CE, as this is a rare event. Second, investigators should aim to compare ONSD measurements to gold standard techniques when feasible. Obtaining elective lumbar puncture for opening pressure measurement in acutely ill children, as well as the time delay from ultrasound measurement to MRI, will pose unique challenges. Third, investigators should examine how the ONSD changes with time during DKA. This is particularly important to explore the relationship between the ONSD and rehydration. Serial measurements of the ONSD during treatment of DKA will be needed, and determining the effect size of specific treatments will likely require a larger sample. Finally, our finding that mean ONSD measurements are above the upper limit of normal in children with T1D should be validated in larger studies and involve a comparison of the coronal and axial imaging techniques.

## CONCLUSION

In our study population, mean ONSD measurement did not vary significantly based on T1D-related illness severity and was not associated with DKA-CE risk factors in our regression model. Our findings suggest that the progression to subclinical disease and continuum of DKA-CE development may not be sufficiently discriminated by this technique. Our results may inform further studies designed to capture patients with mild neurologic injury who are more likely to develop subclinical DKA-CE, and those with clinically overt disease. Other areas for future investigation include assessment of serial measurements of the ONSD during rehydration and correction of acidosis using a paired analysis, and serial evaluation of the ONSD during the peak onset of DKA-CE (4–12 hours into therapy). Multi-center efforts will likely be needed to capture patients with subclinical or clinically overt DKA-CE.

## Supplementary Information



## Figures and Tables

**Table 1:** Demographic and clinical characteristics of study patients. Control patients were enrolled in the Endocrine Clinic, and DKA and non-DKA patients were enrolled in the emergency department.

Demographics	Control (n=36)	Non-DKA (n=36)	DKA (n=36)	p-value
Age, y	12.0 (9.5–15.0)	12.0 (10.0–14.5)	13.0 (11.5–14)	0.86
Gender, male	20 (56)	19 (53)	22 (61)	0.77
Race*				0.22
White	34 (94)	31 (86)	27 (75)	
African-American	2 (6)	3 (8)	6 (17)	
Ethnicity				0.36
Hispanic/Latino	1 (3)	0 (0)	2 (6)	
Non-Hispanic/Latino	35 (97)	36 (100)	34 (94)	
History of T1Dξ				0.81
New-onset	–	21 (58)	22 (61)	
≥1 year	23 (64)	13 (36)	13 (36)	
Past DKA events				0.38
0	30 (83)	27 (75)	25 (69)	
≥1	6 (17)	9 (25)	11 (31)	

Data reported as *n* (%) or median (IQR).

*DKA*, diabetic ketoacidosis; *T1D*, type 1 diabetes

* Two patients in the non-DKA group identified as Hawaiian or other Pacific Islander race, one with DKA identified as American Indian or Alaskan Native, and two with DKA identified as Asian.

ξ Well-controlled excluded; all patients in this group had known history of T1D.

**Table 2:** Emergency department evaluation.

Characteristics	Non-DKA (n=36)	DKA (n=36)	p-value
ED evaluation			
Length of symptoms, days	7 (1–10)	5 (1–14)	0.89
Transferred to ED	15 (42)	19 (53)	0.35
Prior IVF (<10 mL/kg)	0 (0)	5 (14)	–
IVF amount*			0.06
≤10 mL/kg	26 (72)	24 (67)	
10–20 mL/kg	4 (11)	1 (3)	
≥20 mL/kg	6 (17)	11 (31)	
Insulin in ED	11 (37)	17 (47)	0.15
% Dehydrationξ	6.0 (2.2–8.8)	8.7 (5.3–14.1)	0.01
Laboratory values			
Sodium	134 (133–136)	135 (132–139)	0.53
Potassium	4.0 (3.7–4.3)	4.4 (3.8–5.0)	0.18
pH	7.36 (7.34–7.39)	7.21 (7.07–7.28)	<0.001
HCO_3_	24 (21–25)	11 (7–14)	<0.001
pCO_2_	39 (34–45)	28 (23–31)	<0.001
BUN	15 (12–17)	16 (12–20)	0.20
Glucose	375 (286–529)	428 (358–620)	0.06
A1C†	12.5 (10.6–14.0)	13.9 (11.9–14.0)	0.11

Data reported as *n* (%) or median (IQR).

*BUN*, blood urea nitrogen; *DKA*, diabetic ketoacidosis; *ED*, emergency department; *HCO_3_*, bicarbonate level; *IVF*, intravenous fluid

* Includes amount of IVF received prior to transfer from another institution. All 5 patients who received IVF prior to transfer ultimately received ≥20 mL/kg of IVF.

ξ One patient in the non-DKA group and 3 in the DKA group did not have follow-up clinic weights to calculate estimated percent dehydration.

† Ten patients in the non-DKA group and 4 in the DKA group did not have hemoglobin A1C drawn in the ED. Our laboratory does not report hemoglobin A1C values >14. Patients with values listed as “>14.0” were considered to be 14 for analysis (T1D with hyperglycemia n=9; DKA n=17).

**Table 3:** Ocular point-of-care ultrasound evaluation.

Characteristic	Control (n=36)	Non-DKA (n=36)	DKA (n=36)	p-value
Time from IVF to US, min*ξ	–	49.0 (20.0–99.5)	74.5 (56.5–136)	0.02
Time to US, min*	–	108 (68–162)	122 (73–191)	0.45
ONSD, mm†	5.2 (0.8)	5.0 (0.9)	5.2 (0.9)	0.79
ONSD, mm*	5.1 (4.5–5.6)	5.0 (4.4–5.5)	5.1 (4.6–5.9)	

* Data reported as median (IQR) or

† mean (SD).

ξ Five patients received IVF prior to ED arrival in the DKA group, and were excluded from analysis.

*DKA*, diabetic ketoacidosis; *IQR*, interquartile range; *IVF*, intravenous fluid; *ONSD*, optic nerve sheath diameter; *SD*, standard deviation; *US*, ultrasound

**Table 4:** Missed eligible compared to enrolled patients.

	Missed eligible		p-value	

Characteristics	Non-DKA (n=47)	DKA (n=21)	Enrolled vs missed non-DKA	Enrolled vs missed DKA
Demographics				
Age, y	13 (9–16)	11 (9–15)	0.65	0.42
Gender, male	18 (38)	8 (38)	0.19	0.09
History of T1D			0.22	0.32
New-onset	21 (45)	10 (48)		
≥1 year	26 (55)	11 (52)		
Past DKA events			0.49	0.56
0	32 (68)	13 (62)		
≥1	15 (32)	8 (38)		
ED evaluation				
IVF amount			0.34	0.23
≤10 mL/kg	27 (58)	12 (57)		
10–20 mL/kg	10 (21)	3 (14)		
≥20 mL/kg	10 (21)	6 (29)		
Insulin in ED	14 (30)	16 (76)	0.94	0.03
% Dehydration*	3.2 (0.3–7.3)	9.1 (4.3–12.6)	0.12	0.77
Laboratory values				
Sodium	134 (131–136)	134 (132–138)	0.37	0.85
Potassium	4.1 (3.8–4.4)	4.5 (4.1–5.0)	0.25	0.34
pH	7.38 (7.36–7.41)	7.20 (7.10–7.29)	0.13	0.47
HCO_3_	23 (21–24)	9 (7–15)	0.26	0.77
pCO_2_	38 (36–42)	27 (23–36)	0.77	0.80
BUN	14 (11–17)	17 (12–20)	0.56	0.83
Glucose	363 (255–494)	492 (384–669)	0.72	0.23
A1Cξ	11.2 (9.5–14.0)	13.9 (11.3–14.0)	0.39	0.63

Data reported as *n* (%) or median (IQR).

*BUN*, blood urea nitrogen; *DKA*, diabetic ketoacidosis; *ED*, emergency department; *HCO_3_*, bicarbonate level; *IVF*, intravenous fluid; *T1D*, type 1 diabetes

* Five patients in the missed non-DKA group and 3 in the missed DKA group did not have follow-up clinic weights to calculate estimated percent dehydration.

ξ Eleven patients in the missed non-DKA group and 3 patients in the missed DKA group did not have hemoglobin A1C levels drawn in the ED.
