# Supplementary material for: Optic Nerve Sheath Diameter Measurement During Diabetic Ketoacidosis: A Pilot Study
Source: West J Emerg Med. 2016 Jul 25;17(5):531–41. doi: 10.5811/westjem.2016.6.29939 (PMC5017836; doi:10.5811/westjem.2016.6.29939)
Supplement: Supplementary file 1 [file wjem-17-531-s001.docx]

| **Supplemental Table 1.** Missed eligible compared to enrolled patients. | | | | | |
| --- | --- | --- | --- | --- | --- |
|  | Missed eligible | | p-value | | |
| Characteristics | Non-DKA (N=47) | DKA  (N=21) | | Enrolled vs  missed non-DKA | Enrolled vs missed DKA |
| Demographics |  | | | | |
| Age, y | 13 (9–16) | 11 (9–15) | | 0.65 | 0.42 |
| Gender, male | 18 (38) | 8 (38) | | 0.19 | 0.09 |
| History of T1D |  |  | | 0.22 | 0.32 |
| New-onset | 21 (45) | 10 (48) | |  |  |
| ≥1 year | 26 (55) | 11 (52) | |  |  |
| Past DKA events |  |  | | 0.49 | 0.56 |
| 0 | 32 (68) | 13 (62) | |  |  |
| ≥1 | 15 (32) | 8 (38) | |  |  |
| ED evaluation |  | | | | |
| IVF amount |  |  | | 0.34 | 0.23 |
| ≤10 mL/kg | 27 (58) | 12 (57) | |  |  |
| 10–20 mL/kg | 10 (21) | 3 (14) | |  |  |
| ≥20 mL/kg | 10 (21) | 6 (29) | |  |  |
| Insulin in ED | 14 (30) | 16 (76) | | 0.94 | 0.03 |
| % Dehydration^*^ | 3.2 (0.3–7.3) | 9.1 (4.3–12.6) | | 0.12 | 0.77 |
| Laboratory values |  | | | | |
| Sodium | 134 (131–136) | 134 (132–138) | | 0.37 | 0.85 |
| Potassium | 4.1 (3.8–4.4) | 4.5 (4.1–5.0) | | 0.25 | 0.34 |
| pH | 7.38 (7.36–7.41) | 7.20 (7.10–7.29) | | 0.13 | 0.47 |
| HCO_3_ | 23 (21–24) | 9 (7–15) | | 0.26 | 0.77 |
| pCO_2_ | 38 (36–42) | 27 (23–36) | | 0.77 | 0.80 |
| BUN | 14 (11–17) | 17 (12–20) | | 0.56 | 0.83 |
| Glucose | 363 (255–494) | 492 (384–669) | | 0.72 | 0.23 |
| A1C**^ξ^** | 11.2 (9.5–14.0) | 13.9 (11.3–14.0) | | 0.39 | 0.63 |
